# Sensory descriptive quantitative analysis of unpasteurized and pasteurized juçara pulp (*Euterpe edulis*) during long-term storage

**DOI:** 10.1002/fsn3.105

**Published:** 2014-05-06

**Authors:** Paula Porrelli Moreira da Silva, Renata Cristina Casemiro, Rafaela Rebessi Zillo, Adriano Costa de Camargo, Evanilda Teresinha Perissinotto Prospero, Marta Helena Fillet Spoto

**Affiliations:** 1 Center for Nuclear Energy in Agriculture, University of São Paulo (CENA/USP)Piracicaba, São Paulo, Brazil; 2 Agri-food Industry, Food and Nutrition Department, Luiz de Queiroz College of Agriculture (ESALQ/USP), University of São Paulo (USP)Piracicaba, São Paulo, Brazil

**Keywords:** Correlation, *Euterpe edulis*, principal component analysis, QDA, sensory profile

## Abstract

This study evaluated the effect of pasteurization followed by storage under different conditions on the sensory attributes of frozen juçara pulp using quantitative descriptive analysis (QDA). Pasteurization of packed frozen pulp was performed by its immersion in stainless steel tank containing water (80°C) for 5 min, followed by storage under refrigerated and frozen conditions. A trained sensory panel evaluated the samples (6°C) on day 1, 15, 30, 45, 60, 75, and 90. Sensory attributes were separated as follows: appearance (foamy, heterogeneous, purple, brown, oily, and creamy), aroma (sweet and fermented), taste (astringent, bitter, and sweet), and texture (oily and consistent), and compared to a reference material. In general, unpasteurized frozen pulp showed the highest score for foamy appearance, and pasteurized samples showed highest scores to creamy appearance. Pasteurized samples remained stable regarding brown color development while unpasteurized counterparts presented increase. Color is an important attribute related to the product identity. All attributes related to taste and texture remained constant during storage for all samples. Pasteurization followed by storage under frozen conditions has shown to be the best conservation method as samples submitted to such process received the best sensory evaluation, described as foamy, slightly heterogeneous, slightly bitter, and slightly astringent.

## Introduction

Juçara palm (*Euterpe edulis*), native of the Brazilian Atlantic Forest, belongs to the genus Euterpe, which comprises 28 species found among the West Indies and South America. High economic value has been attributed Juçara palm because of its heart palm extraction, which is well appreciated in gastronomy. Furthermore, the use of its seed and pulp has been increasing in the few last years. Juçara pulp is similar to the pulp of açai (*Euterpe oleracea*), which is already well accepted by consumers (Favreto 2010).

Açai and juçara pulp have strong reddish-purple coloration due to the presence of antioxidants belonging to anthocyanins, a polyphenol subclass of flavonoids. According to Degáspari and Waszczynskyj (2004), fruits with such color are good sources of anthocyanins. However, juçara pulp can deteriorate easily. A few hours at room temperature can change its characteristics due to the action of microorganisms and enzymes as well as oxidation. To avoid detrimental effects, it is necessary to apply treatments to improve its conservation. Pasteurization inactivates enzymes and decrease or eliminates spoilage and pathogenic microorganisms through heating, which improves the shelf life of the product (Pereda 2005).

Descriptive sensory analysis is a sophisticated method when compared with the methods of discrimination and acceptance. The results of sensory descriptive analysis testing offer a complete description of the product, that is, the basis for mapping the similarities and disparity of samples. The results enable us to make correlations between ingredients or production process variables with the sensory attributes (Stone and Sidel 2004). Future sensory tests of juçara pulps can be included among the quality assurance of the product, because it is a multidimensional measure integrated capable of quickly detecting sensory particularities that cannot be detected by other analytical procedures and still be able to determine whether a product is well accepted or not (Muñoz et al. 1992).

To the best of our knowledge, there is no information regarding the sensory evaluation of juçara pulp subjected to pasteurization followed by storage under different conditions. Such as with other conservation methods, pasteurization may be detrimental to sensory attributes, thus sensory evaluation is necessary to evaluate the impact of the process. Furthermore, there is a growing demand for juçara pulp in southeastern Brazil. Given the huge distances that the product must move to get to the consumers, increasing its shelf life is mandatory. Additionally, increasing the shelf life of the product will allow for exportation, causing a profit increase to the food industry and being beneficial to consumers. Thus, the aim of this study was to evaluate the effect of pasteurization and storage under refrigerated and frozen conditions on the sensory attributes juçara pulp by applying quantitative descriptive analysis (QDA).

## Materials and Methods

### Materials

Juçara palm fruits, harvested in the city of Mogi das Cruzes, SP, Brazil, were selected, washed with water, and sanitized with NaDCC dihydrate (sodium dichloro-s-triazinetrione dihydrate) at 200 mg L^−1^ for 15 min, followed by immersion in water at 40°C for 20 min in a stainless steel tank Mecamau (Espírito Santo do Pinhal, SP, Brazil). The pulp was prepared with water (2/1; w/v), using a stainless steel pulper “Bonina Compacta” (Itabuna, BA, Brazil).

The control (unpasteurized sample) was packed in transparent polyethylene bags (100 mL) and stored at frozen condition (−18°C). The remaining samples were packed in laminated multilayer bags (100 mL), composed for 15 *μ* PET (polyethylene terephthalate), 15 *μ* PA (polyamide), and 70 *μ* PP (polypropylene), followed by pasteurization.

Temperature control of pasteurization was performed using thermocouple (Novus, My PC lab, Porto Alegre, RS, Brazil) coupled to four sensors. The bags were immersed in a stainless steel tank with water at 80°C. Samples were kept in the tank for 5 min after internal temperature equilibrium. The product was cooled using a container with cold water until 30°C (internal temperature) and the storage was performed at two different temperatures (6°C and −18°C). Sensory evaluation was performed at the 1, 15, 30, 45, 60, 75, and 90th days of storage. Microbiological analyses were performed in all samples to ensure their safety.

### Raw material characterization

The raw material (frozen pulp) characterization was performed through analysis of pH using a potentiometer Marconi, MA-522 (Piracicaba, SP, Brazil), according to method no. 981.12 from AOAC (2005). Soluble solids content was quantified using the method 932.12 (AOAC 2005) and the results were expressed in °Brix. The moisture content was evaluated using the gravimetric method 950.46 (AOAC 2005) and the results were expressed as moisture percentage.

### Microbiological analysis

Coliform bacteria and *Salmonella* spp. were evaluated to fit the Brazilian food legislation (Brazil 2001).

#### Coliform bacteria

The presence of coliform bacteria was investigated using a multiple-tube fermentation method as described by Downes and Ito (2001). The results were expressed as most probable number (MPN).

#### Salmonella spp

The contamination by *Salmonella* spp. was evaluated using the 1–2 Test for *Salmonella* purchased from Biocontrol (Bio Control, Campinas, SP, Brazil), which is a commercial kit test approved by AOAC (2000). All analyses were performed as recommended by the supplier and the results were reported as presence or absence.

### Sensory analysis—QDA

This study was approved by the Ethics Committee of Human Research of the College of Agriculture “Luiz de Queiroz,” University of São Paulo (ESALQ/USP).

**Figure 1:**
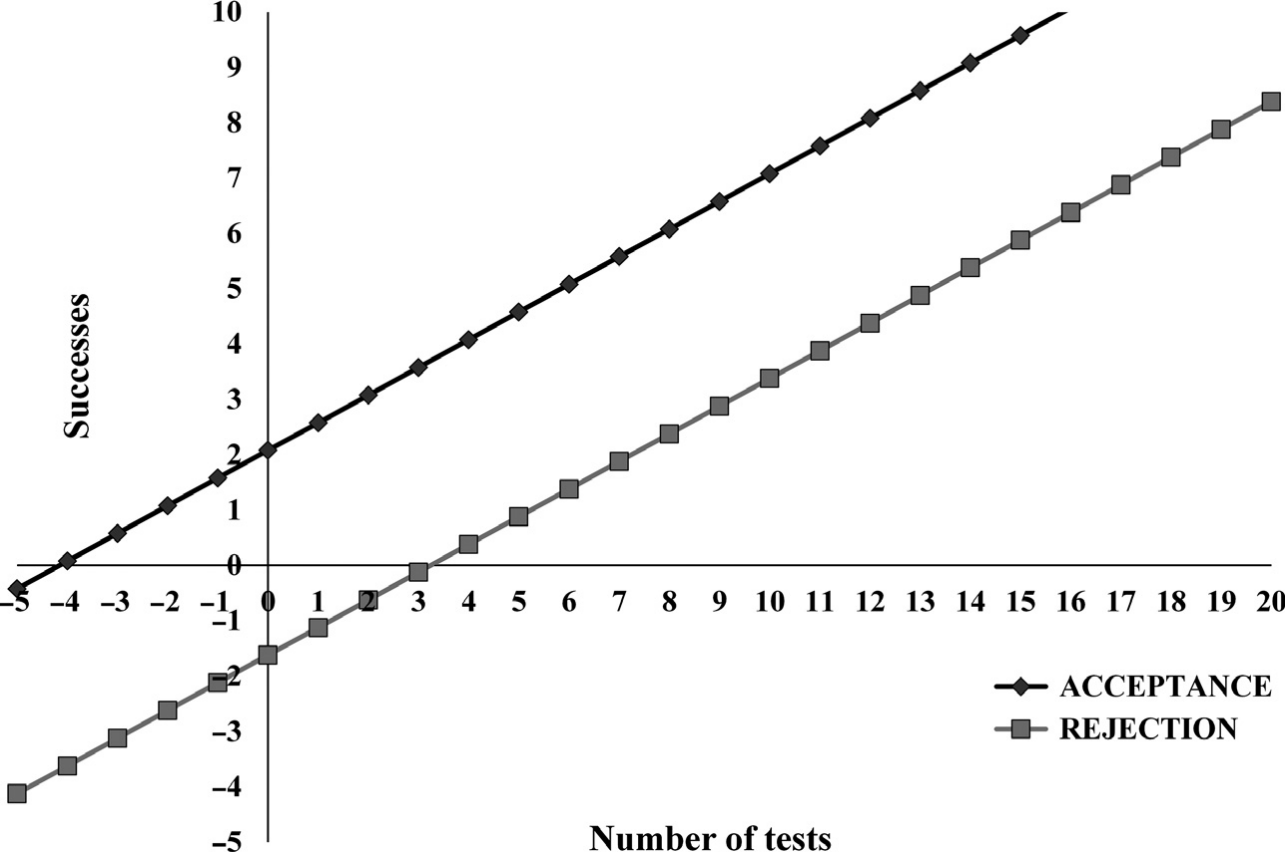
Sequential test triangular to selection of tasters with p0 = 1/3, p1 = 2/3, *α* = 0.05 and *β* = 0.10.

The method used for the sensory evaluation was an adaptation of QDA developed by Stone (1992). The panelists were selected by the basic taste test according to Stone et al. (1998). Candidates who obtained 100% accuracy were chosen for the next stage of selection. In the second stage of selection the sensitivity test was used for taste, using the triangular test, at which the members received three samples with different sucrose concentration, two identical (2%) and one different (4%). Individuals were asked to try the samples from left to right and identify the different one (Meilgaard et al. 2006). Each member performed the test seven times. The final panelists were chosen based on whether their answers fit within the region of acceptance or rejection (Fig. 1), according to Amerine et al. (1965).

As part of the first stage of QDA of juçara pulp, 21 candidates were recruited. Of these, 20 were approved through the test of basic tastes. Only those who accomplished 100% accuracy were approved. After approval in the basic taste test, the panelists took the sensitivity taste test, which uses the triangular test (sequential analysis) with samples containing different contents of sucrose (concentration of 2% and 4% sugar). This method was applied seven times for members of the panel, at the same conditions described for the test of basic tastes, in order to construct the graph referring to the sequential triangular test (Fig. 1), obtaining the lines of acceptance (A) and rejection (R) for each one. The responses of approved individuals were located in the zone of acceptance of the sequential analysis graph (Fig. 1). Sixteen panelists were approved, but only 10 of them went to the next step called survey of descriptors.

#### Determination of relevant attributes

Two representative samples were offered to the final panel to determine relevant attributes: spray-dried juçara pulp reconstituted in a solution of sucrose/water 10% (w/v) and homogenized juçara pulp (without sugar). The test was performed at the same conditions of the basic taste test performed previously, and using the network method (Meilgaard et al. 2006). All members were asked to take notes of the attributes perceived for appearance, aroma, taste, and texture of the samples.

**Figure 2:**
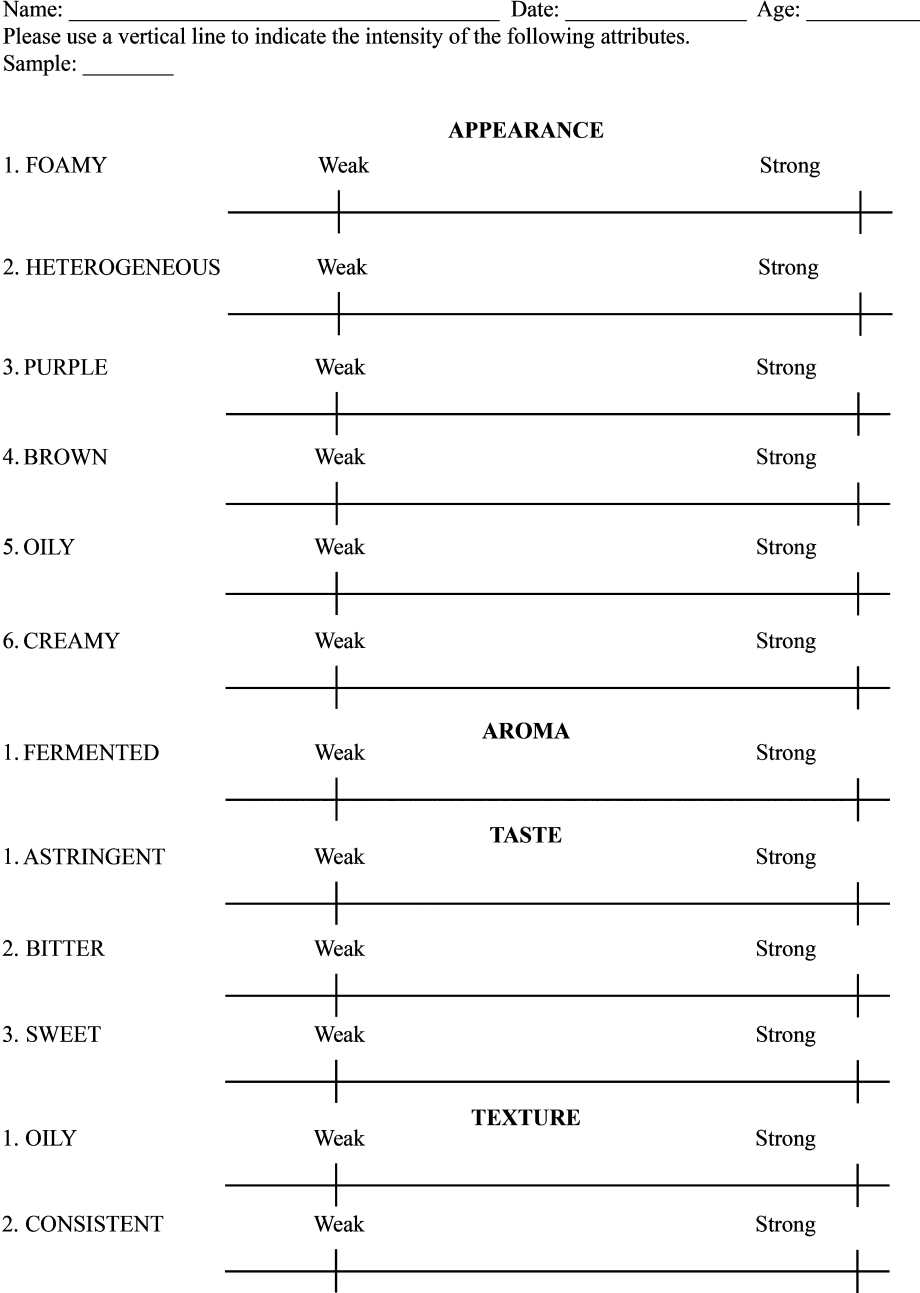
Data sheet used at the final analysis with the intensity scales for each attribute.

The attributes described by the panelists were compiled by the sensory analyst and grouped in similar clusters. Terms that described best the samples were used for preparation of a preliminary lexicon used for further training of the panelists (Table 2). The outliers were eliminated (Damásio and Costell 1991) and a ballot containing all attributes consisting of unstructured scale (10 cm) ranging from weak (note 1) to strong (note 9) was developed (Fig. 2).

#### Training

The training was performed with three different samples in order to ensure that all attributes would be noticed: sample 1, consisting of homogenized pulp; sample 2, consisting of pulp added by sugar (10% w/v); and sample 3, consisting of spray-dried pulp reconstituted with water (10% w/v). Panelists were asked to evaluate the intensity of each attribute of the three samples using the evaluation form previously developed by the sensory analyst (Fig. 2). The form containing definitions about the reference material (Table 2) as well as the actual reference material was shown to the panelist during the sessions.

The training was performed in three sessions, until it was verified that all members were able to perform the tests and that their answers would be trustworthy (Moraes 1993).

#### Evaluation of panelists' performance

The samples were served at 6°C in plastic cups coded with three-digit numbers. Panelists were asked to rinse their mouth with water between each sample. Each member received the same sample in triplicate without knowledge of its nature to ensure the quality of the results. This step was conducted to evaluate if the panelist would be able to produce statistically significant data.

Results of each panelist and each attribute were submitted to statistical analysis of variance (ANOVA) with two sources of variation (sample and repetition). Panelists who demonstrated ability at discriminating attributes (pF_samples_ ≤ 0.3), and repeatability in the evaluation (pF_repetitions_ ≥ 0.05), as well as consensus with other members were selected for the final team (Damásio and Costell 1991).

#### QDA of samples

The samples of juçara pulp (frozen unpasteurized—F; pasteurized after packing and stored at refrigerated condition—PR, and pasteurized after packing stored at frozen condition—PF) were evaluated at the 1, 15, 30, 45, 60, 75, and 90th days. All tests were performed in individual cabins with white light. The samples were served in three-digit coded plastic cups (50 mL), at 6°C, without any sugar.

#### Statistical analysis

The experimental design was completely randomized with seven repetitions. The results were evaluated using the Statistical Analysis System 9.2 (Statistical Analysis System Institute 1996) and submitted to ANOVA. The statistical difference of means was determined by Tukey's test (*P* < 0.05). Data were also submitted to multivariate statistical and mathematical treatments using the software PC-ORD (McCune and Mefford 1999).

## Results and Discussion

### Raw material characterization

Analyses of pH and total soluble solids (TSS) of juçara pulp (Table 1) indicated that the product is susceptible to degradation. The combination of high pH with TSS at low levels make the product perishable (Menezes et al. 2008). As the product presented such characteristics, the application of a conservation method is mandatory. Furthermore, juçara pulp has a high moisture content, which makes the product susceptible to microorganism proliferation.

**Table 1:** pH, Soluble solids content, titratable acidity, and moisture of juçara pulp frozen (mean values ± SD, *n* = 6)

Variables	Results
pH	4.38 ± 0.01
Soluble solids content (°Brix)	2.81 ± 0.12
Moisture (%)	89.4 ± 2.38

SD, standard deviation of the mean; n, number of replicates.

**Table 2:** Definition and reference material for each attribute

Attribute	Definition	Reference
Appearance		
Foamy	Juçara pulp homogenized in a blender, with bubbles.	*Strong:* pulp homogenized for 2 min in a blender with air bubbles.
		*Weak:* unhomogenized pulp, without air bubbles.
Heterogeneous	Juçara pulp nonuniform (color, bubbles, granules, oil droplets, stains).	*Strong:* juçara pulp added by coffee powder.
		*Weak:* filtered juçara pulp.
Purple	Juçara pulp at ideal conditions of consumption (nonoxidized).	*Strong:* juçara pulp added by a 5% solution of citric acid followed by freezing.
		*Weak:* juçara pulp stored at room temperature for 24 h and exposed to oxygen.
Brown	Juçara pulp stored at room temperature and exposed to the action of oxygen.	*Strong:* juçara pulp stored at room temperature for 24 h and exposed to the action of oxygen.
		*Weak:* juçara pulp added by a 5% solution of citric acid followed by freezing.
Oily	Juçara pulp with oil droplets.	*Strong:* juçara pulp with oil droplets.
		*Weak:* juçara pulp added by 50% of water.
Creamy	Appearance of viscous, thickened.	*Strong:* juçara pulp plus corn starch.
		*Weak:* juçara pulp added by 50% of water.
Aroma		
Fermented	Juçara pulp stored for a period and temperature to induce fermentation.	*Strong:* juçara pulp added by biological yeast.
		*Weak:* juçara pulp added by 50% of water.
Taste		
Astringent	Juçara pulp with taste that induces the tongue's constriction.	*Strong:* juçara pulp added by 0.5% tannin.
		*Weak:* juçara pulp added by 50% of water.
Bitter	Juçara pulp with a taste that is sharp, acrid, and unpleasant.	*Strong:* juçara pulp added by 0.1% of caffeine solution.
		*Weak:* juçara pulp added by 50% of water.
Sweet	Juçara pulp with the taste of honey or sugar.	*Strong:* juçara pulp added by 20% sucrose.
		*Weak:* juçara pulp plus 50% water.
Texture		
Oily	Juçara pulp with sensation of oil in the mouth.	*Strong:* juçara pulp added by 5% of soybean oil.
		*Weak:* juçara pulp added by 50% of water.
Consistent	Thickened, difficult to drain.	*Strong:* juçara pulp added by corn starch.
		*Weak:* pure juçara pulp.

The juçara pulp has a humidity of ∼90% (Table 1) because the content amount of water in some fruit pulp is determined at pulping be taking into account the desired dry matter content (Silva et al. 2013).

The proximate and fatty acid composition of juçara pulp was already reported (Silva et al. 2013). The proximate composition analysis demonstrated carbohydrates as the major component, followed by lipid and protein. Additionally, the major fatty acids were 36.0% of oleic acid, 34.4% of palmitic acid, and 19.2% of linoleic acid, which gives 55.2% of unsaturated fatty acids.

### Microbiological analysis

The microbiological evaluation indicated absence of *Salmonella* spp. and <0.3 MPN of coliform bacteria, thus the samples fit the legal standards of the Brazilian Food Laws (Brazil 2001).

### Sensory analysis

#### Survey and grouping of attributes

The evaluated attributes were grouped according to the panel consensus (Table 2), including six for appearance (foamy, heterogeneous, purple, brown, oily, creamy), two for aroma (sweet, fermented), three for taste (astringent, bitter, sweet) and two for texture (oily, consistent). A paper ballot was developed with intensity scales for each attribute (Fig. 2).

#### Training and evaluation of the performance of panelists

After three training sessions, and based on the exclusion criteria defined by Damásio and Costell (1991), seven panelists were selected to be part of the sensory team for the final evaluation. They were individuals that presented at least 80% of the evaluation in consensus with the sensory team. The final sensory team was composed of women (100%) aged between 20 and 25 years.

#### Quantitative descriptive analysis

The QDA was carried out with samples at 6°C for pulp pasteurized and stored under refrigerated condition (6°C) (PR), pulp pasteurized and stored at frozen condition (−18°C) (PF), and pulp unpasteurized and stored at frozen condition (−18°C) (UF). All results are shown in Table 3. Due to their deteriorated appearance after some storage time, the sensory evaluation for the taste and texture of the PR was performed only on days 1 and 15 after processing.

**Table 3:** Appearance attributes of juçara pulp submitted to different conservation and storage conditions (mean values ± SD, *n* = 7)

Treatments	Periods (days)						

	1	15	30	45	60	75	90
Foamy							
UF	5.28 ± 3.01 Aa	4.57 ± 2.31 ABa	5.08 ± 1.66 ABa	4.03 ± 2.70 ABa	5.91 ± 1.69 ABa	7.13 ± 1.69 ABa	2.77 ± 1.99 Ba
PR	2.64 ± 1.04 Aa	3.11 ± 1.83 Aa	2.57 ± 0.83 Aa	3.36 ± 1.60 Aa	4.99 ± 1.61 Aa	2.34 ± 1.31 Ab	2.74 ± 1.76 Aa
PF	2.53 ± 1.40 Aa	4.45 ± 1.77 Aa	3.17 ± 1.24 Aa	3.26 ± 1.32 Aa	3.46 ± 2.14 Aa	4.73 ± 1.54 Aab	5.41 ± 1.82 Aa
Purple							
UF	5.37 ± 2.62	5.27 ± 2.69	5.06 ± 2.06	5.60 ± 1.18	3.58 ± 2.18	1.80 ± 1.40	2.70 ± 2.60
PR	2.38 ± 1.18	2.71 ± 1.51	3.63 ± 2.05	2.38 ± 1.42	2.66 ± 2.34	2.83 ± 2.71	2.20 ± 1.88
PF	2.38 ± 1.39	2.61 ± 1.45	3.24 ± 1.77	2.24 ± 1.39	2.56 ± 2.24	2.57 ± 2.20	2.13 ± 1.80
Brown							
UF	4.64 ± 2.54 BCa	4.40 ± 2.36 Ca	5.57 ± 1.07 ABCa	3.23 ± 1.79 Cb	5.57 ± 2.03 ABCa	8.46 ± 1.65 Aa	8.13 ± 2.03 ABa
PR	7.50 ± 1.89 Aa	6.86 ± 2.60 Aa	7.43 ± 1.30 Aa	7.21 ± 1.12 Aa	7.33 ± 2.00 Aa	6.41 ± 2.28 Aa	8.53 ± 1.27 Aa
PF	7.84 ± 1.46 Aa	6.66 ± 2.73 Aa	7.30 ± 1.15 Aa	7.31 ± 1.13 Aa	7.09 ± 2.10 Aa	6.78 ± 2.19 Aa	8.75 ± 1.12 Aa
Oily							
UF	4.00 ± 2.64	3.93 ± 2.50	4.21 ± 2.63	4.29 ± 2.19	4.07 ± 2.35	5.88 ± 1.79	5.93 ± 2.54
PR	5.27 ± 3.07	5.10 ± 2.36	3.74 ± 2.32	4.96 ± 201	4.54 ± 2.91	4.31 ± 2.78	5.38 ± 2.36
PF	5.16 ± 2.75	4.43 ± 1.88	5.28 ± 1.70	4.91 ± 2.18	4.91 ± 2.36	4.67 ± 2.79	5.73 ± 1.54
Creamy							
UF	7.56 ± 0.88 Aa	5.50 ± 1.98 ABa	6.49 ± 1.32 ABa	4.57 ± 1.88 ABa	6.64 ± 2.35 ABa	3.91 ± 2.16 Ba	6.00 ± 1.41 ABa
PR	6.61 ± 1.90 Aa	5.73 ± 2.80 Aa	6.78 ± 0.98 Aa	5.61 ± 1.10 Aa	5.90 ± 1.85 Aa	6.43 ± 1.33 Aa	6.24 ± 1.12 Aa
PF	6.34 ± 2.33 Aa	5.94 ± 1.95 Aa	6.76 ± 0.53 Aa	5.06 ± 1.81 Aa	6.29 ± 1.03 Aa	5.96 ± 1.29 Aa	6.37 ± 1.29 Aa
Heterogeneous							
UF	3.81 ± 1.60	3.89 ± 1.60	3.34 ± 2.38	3.54 ± 2.14	3.93 ± 2.53	5.74 ± 2.65	4.11 ± 1.54
PR	3.23 ± 1.64	4.23 ± 2.35	2.57 ± 1.14	3.19 ± 1.23	3.67 ± 2.04	3.79 ± 2.47	4.17 ± 2.47
PF	3.36 ± 2.12	4.57 ± 1.81	2.88 ± 1.26	3.51 ± 1.45	3.56 ± 2.10	3.50 ± 1.75	4.76 ± 2.64

Means followed by the same capital letter in the row and lower case in the column are not statistically different by Tukey's Test (*P* > 0.05). SD, standard deviation of the mean; n, number of replicates; UF, unpasteurized juçara pulp stored at frozen conditions; PR, pasteurized juçara pulp stored at refrigerated conditions; PF, pasteurized juçara pulp stored at frozen conditions.

Over the storage time, no statistical difference was observed for the foamy attribute in both samples (PF and PR). The samples received mean scores of 3.11 and 3.86, respectively. Only PR showed a significant difference (*P* < 0.05) in relation to UF at the 75th day of storage. However, in general, no statistical difference was observed among treatments regarding the foamy attribute. No statistical difference (*P* < 0.05) was found among treatments and storage time regarding the purple appearance, which was classified as weak purple appearance receiving the mean score of 2.61 (Table 3). Furthermore, during storage time an increase was observed in the brown attribute of the UF sample (*P* < 0.05), which may be due to the degradation of anthocyanins present in the pulp over the storage time (Skrede et al. 2000). On the other hand, PR and PF remained stable regarding the brown attribute during the storage time. Additionally, only at the 45th day did pasteurized samples present statistical difference when compared to the UF sample. Thus, it can be inferred that, in general, no detrimental effects were observed due to pasteurization for color parameters of appearance (purple and brown). Finally, no statistical difference was observed for the oily appearance due to storage time or sample treatment (*P* > 0.05). The samples received a mean score of 3.75, belonging to an intermediate level of oiliness. The same was observed for the creamy appearance, and the samples received a mean score of 6.14, being classified as “strong creamy.” As observed for appearance attributes the creamy one was not affected by pasteurization and, in general, no effect was observed due to the storage time. The heterogeneous appearance and the fermented aroma attribute were not affected by the storage time nor the treatments. The samples received a mean score of 3.77 for heterogeneous appearance, and a score of 4.0 for fermented aroma attribute; both scores classify the products into an intermediate level for each attribute.

The taste-related attributes (astringent, bitter, and sweet) were not affected by storage time or treatments (*P* > 0.05). The astringent taste received a mean score of 5.27, which is close to the midpoint of the unstructured scale. This may be due to the presence of polyphenols in the product, in particular anthocyanins belonging to the flavonoid class. According to Rufino et al. (2010) juçara pulp contains 192 mg eq. cyanidin-1 100 g pulp. The astringency perceived by the panelist is produced by a variety of oro-chemical stimulations related to the presence of polyphenols, acids and aluminum salts. For most of the fruits, this perception is associated to presence of phenolic compounds (Lesschaeve and Noble 2005; Jaeger 2006). Condensed tannins (proanthocyanidins), another subclass of polyphenols present in peanut skins (de Camargo et al. 2012) as well as in grape and wine also confer an astringent mouth taste.

The bitter taste attribute received a mean score of 5.36, indicating an intermediate level of bitterness, which might be considered negative for the consumers. However, juçara pulp is commonly consumed with guarana syrup or fruits, which results a sensory combination appreciated by consumers. All samples were classified as having a weak sweet taste, receiving a mean score of 2.21.

The samples received a mean score of 4.5 for oiliness (Table 4) that was not affected by the storage time nor by the treatments (*P* > 0.05). Menezes (2011) evaluated the attributes of acai pulp and reported higher scores for the oiliness. According to the author, this sensation was related to the high lipid content of the product. This composition influences the sensory aspect; lipids add a mouth feel to the pulp and are useful to produce emulsions in the homogenized product.

**Table 4:** Texture of juçara pulp submitted to different conservation and storage conditions (mean values ± SD, *n* = 7)

Treatments	Periods (days)						

	1	15	30	45	60	75	90
Oily							
UF	3.14 ± 2.03	4.73 ± 2.16	4.47 ± 2.40	4.67 ± 2.13	4.06 ± 2.59	6.04 ± 2.39	4.64 ± 3.15
PR	3.70 ± 2.83	4.86 ± 3.12	–	–	–	–	–
PF	3.75 ± 2.73	5.14 ± 2.45	4.76 ± 2.70	4.56 ± 2.89	4.13 ± 2.55	4.53 ± 2.44	4.87 ± 2.99
Consistent							
UF	5.71 ± 1.58 ABa	4.00 ± 2.46 ABa	6.41 ± 1.07 Aa	3.11 ± 1.90 Ba	5.34 ± 2.20 ABa	4.20 ± 2.02 ABa	5.73 ± 1.42 Aa
PR	4.94 ± 1.59 Aa	6.73 ± 1.45 Aa	–	–	–	–	–
PF	5.50 ± 1.45 Aa	5.99 ± 1.00 Aa	6.60 ± 0.62 Aa	4.73 ± 1.70 Aa	5.14 ± 1.84 Aa	5.71 ± 1.93 Aa	5.56 ± 1.53 Aa

Means followed by the same capital letter in the row and lower case in the column are not statistically different by Tukey's Test (*P* > 0.05). SD, standard deviation of the mean; n, number of replicates; UF, unpasteurized juçara pulp stored at frozen conditions; PR, pasteurized juçara pulp stored at refrigerated conditions; PF, pasteurized juçara pulp stored at frozen conditions.

Regarding consistent texture, no effect was evidenced by the treatment or storage time in comparison to the control (UF, day 1). Pasteurized pulps received a score of 5.65, at the midpoint of the unstructured scale (Table 4).

#### Sensory profile of samples

**Figure 3:**
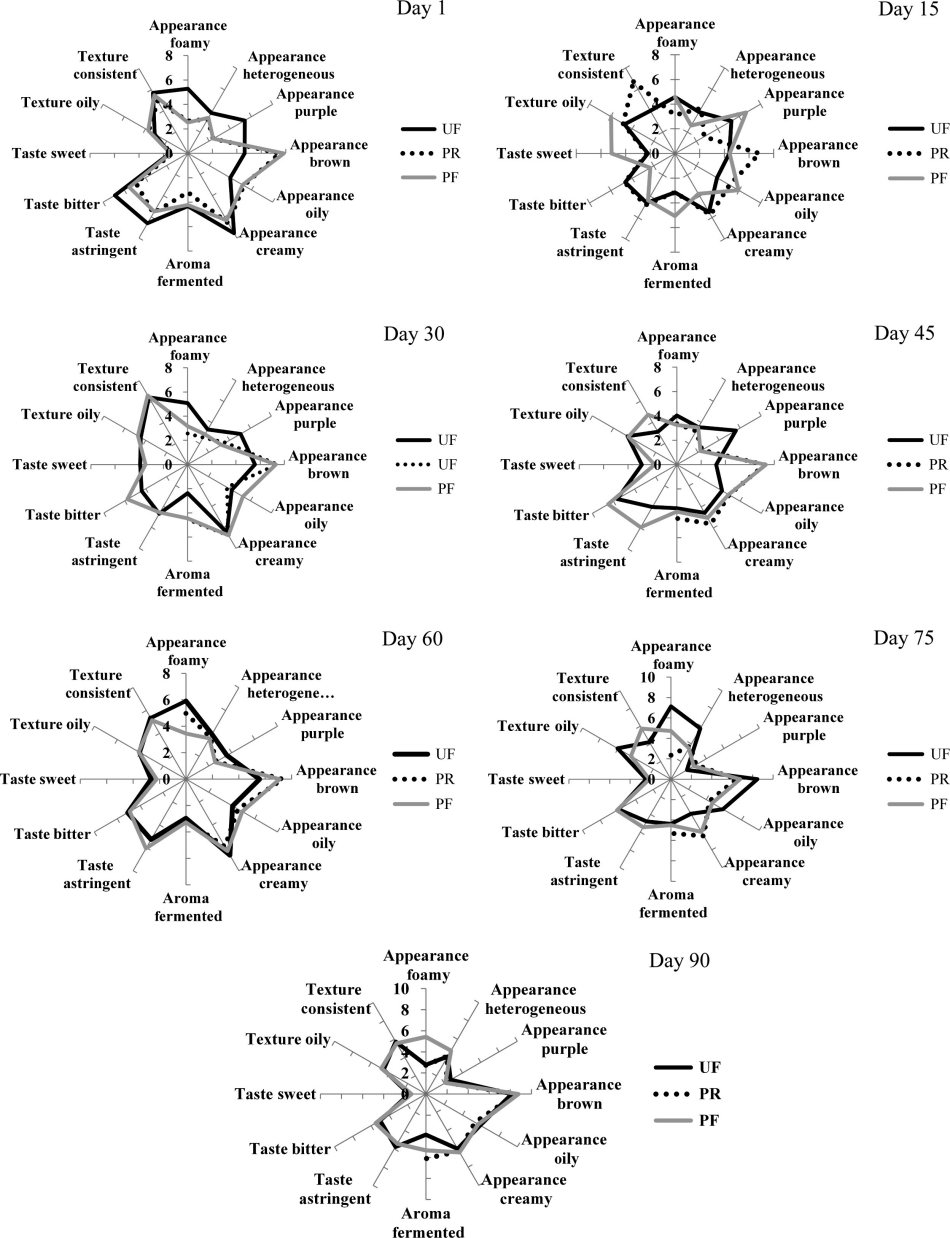
Sensory profile of juçara pulp samples submitted to different conservation methods and storage conditions.

The present data were used to develop the sensory profile of juçara pulp submitted to different conservation methods and storage conditions (Fig. 3). Each sensory descriptor or attribute evaluated was represented by an axis that starts at the center of the figure, which is the point zero of the scale. The intensity of each descriptor increases in the direction of the edge of the graph. The mean score received for each attribute for a particular treatment is presented at the corresponding axis. The sensory profile of each sample was drawn at the point of connection represented by the mean score for each descriptor (Stone et al. 1998; Santana et al. 2006).

At the first day of evaluation, there was no difference between pasteurized samples (PR and PR). The samples received highest scores for foamy and heterogeneous (appearance), sweet and astringent (aroma), and bitter (taste), while unpasteurized samples presented higher scores for purple color when compared to their pasteurized counterparts.

At day 15 there was an increase in the consistent attribute of the pasteurized samples, while a decrease was observed for the unpasteurized one. Unpasteurized samples also presented a decrease at the purple color (30 and 45 days of storage). However, the scores were still higher than that of the pasteurized samples. For the fermented aroma and bitter taste, the unpasteurized sample presented the best sensory evaluation. From day 60 of evaluation, no difference among treatments or storage time was noticed.

#### Principal component analysis of the samples and sensory attributes

**Figure 4:**
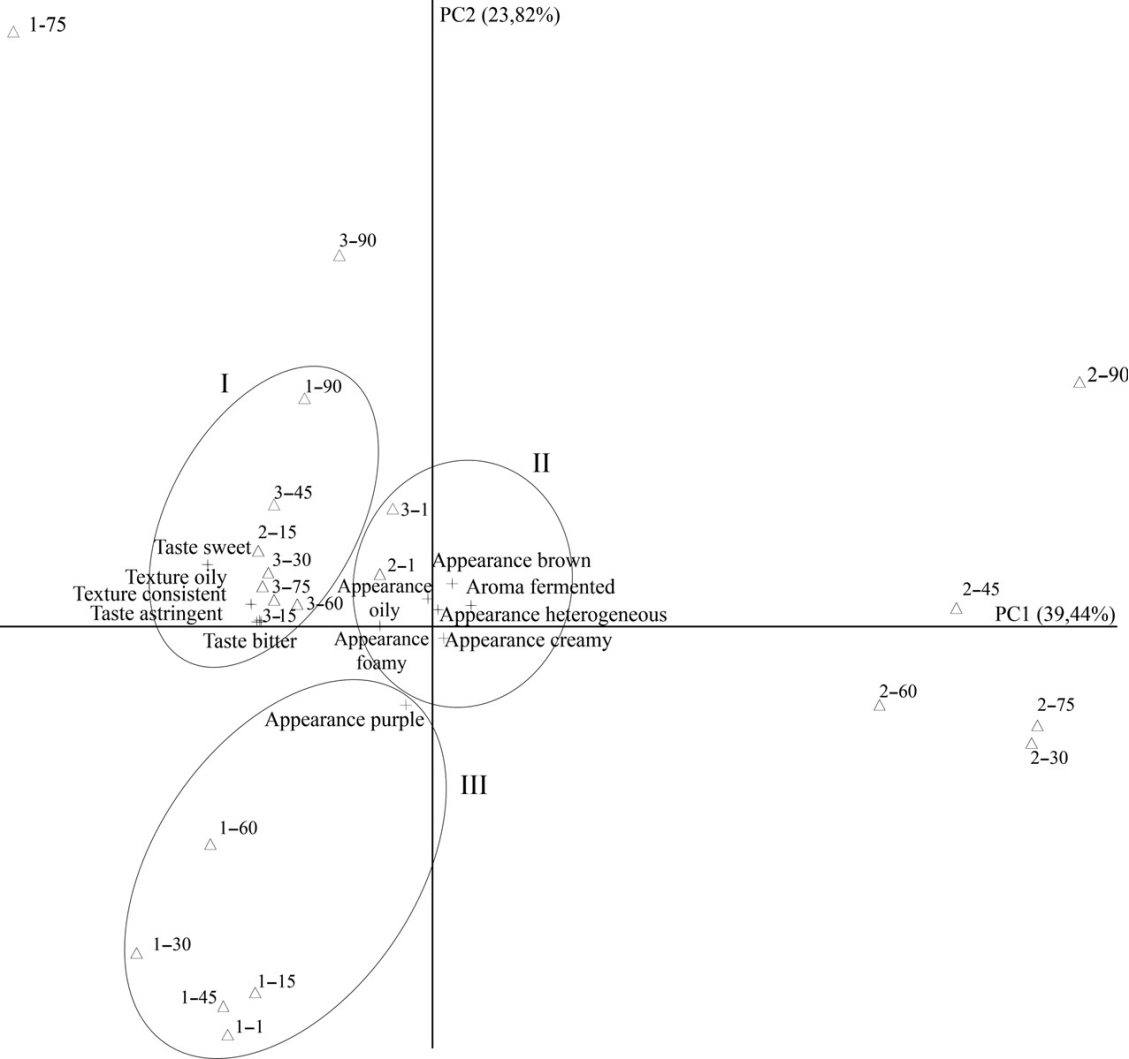
Principal components of the sensory attributes of juçara pulp samples during long-term storage.

Two principal components were generated with the data, explaining 63.26% of the variance. The first principal component (PC1) explained 39.44% of the statistical variance showing positive values for attributes of brown, heterogeneous, and creamy appearances as well as for fermented aroma. Negative values were verified for oily, foamy, and purple appearances; sweet, astringent, and bitter tastes as well as for consistent and oily textures (Fig. 4).

The second principal component (PC2) explained 23.82% of the statistical variance, having positive values for: sweet, astringent, and bitter taste; oily and consistent texture; brown, heterogeneous, oily, and foamy appearances; and fermented aroma. Finally, negative values were observed for creamy and purple appearances (Fig. 4).

The principal components of the samples were plotted in a graph and can be divided into three main groups according to the storage time for each one (Fig. 4). Group I represents frozen unpasteurized samples evaluated on the day 90, pasteurized frozen samples stored from day 15 to 75; and pasteurized samples stored under refrigerated conditions evaluated at day 15. This group was characterized by the sweet, astringent, and bitter taste, as well as for the oily and consistent texture. This datum supported the findings discussed before as evaluated by Tukey's tests. Furthermore, it was demonstrated that pasteurization followed by storage at frozen conditions not only influenced the sensory evaluation but also conferred an identity to the processed food product.

Group II represents pasteurized samples stored at refrigerated or frozen conditions at their first day of sensory evaluation. Positive scores were attributed for oily and foamy appearance. Brown, heterogeneous, and creamy appearance as well as fermented aroma had little influence in the samples of this group. The creamy appearance was not considered as representative for the samples.

Unpasteurized frozen samples stored from day 1 to 60 fit into group III, characterized by the purple color appearance (Fig. 4).

These results show the similarity between the pasteurized samples, regardless of the storage temperature. Furthermore, a clear difference was noticed between pasteurized and unpasteurized frozen samples.

## Conclusion

Regarding appearance attributes, this study demonstrated that, in general, unpasteurized samples were less stable during long-term storage, while pasteurized ones did not change over the time. Pasteurization followed by storage at refrigerated conditions should not be considered for further industrial application as such treatment was not efficient at keeping the sensory attributes of the samples. In comparison, pasteurization followed by frozen storage retained the sensory attributes of texture and taste. This treatment did not affect appearance, texture, and taste attributes. Because of this, it is recommended for industrial application.

Unpasteurized and pasteurized samples followed by frozen storage were reported by the panelists as foamy, slightly heterogeneous, slightly bitter tasting, and slightly astringent. Such descriptors will be helpful for food technologists when developing new products containing juçara pulp.
